# Kidney-Based Molecular Diagnostics of Freshwater Drowning: Comparative Evaluation of SP-A2 and AQP5 in an Animal Model

**DOI:** 10.3390/diagnostics16193086

**Published:** 2026-09-23

**Authors:** Emina Dervišević, Hedim Osmanović, Teufik Goletić, Pietro Zuccarello, Ilma Terzić, Adis Softić, Zurifa Ajanović, Aida Bešić, Francesco Sessa

**Affiliations:** 1 Department of Forensic Medicine, Faculty of Medicine, University of Sarajevo, 71000 Sarajevo, Bosnia and Herzegovina; emina.dervisevic@mf.unsa.ba; 2 Faculty of Natural Sciences and Mathematics, University of Tuzla, Urfeta Vejzagica 4, 75000 Tuzla, Bosnia and Herzegovina; hedim@bih.net.ba; 3 Veterinary Faculty, University of Sarajevo, 71000 Sarajevo, Bosnia and Herzegovina; teufik.goletic@vfs.unsa.ba (T.G.); ilma.terzic@vfs.unsa.ba (I.T.); adis.softic@vfs.unsa.ba (A.S.); 4 Department of Psychology and Health Sciences, Faculty of Human Sciences, Education, and Sports, Pegaso Telematic University, 80143 Naples, Italy; pietro.zuccarello@unipegaso.it; 5 Department of Human Anatomy, Faculty of Medicine, University of Sarajevo, 71000 Sarajevo, Bosnia and Herzegovina; zurifa.ajanovic@mf.unsa.ba; 6 Department of Clinical Sciences Veterinary Medicine, Veterinary Faculty, University of Sarajevo, 71000 Sarajevo, Bosnia and Herzegovina; katicaaida1@gmail.com

**Keywords:** drowning diagnostics, forensic pathology, drowning, kidney tissue, biomarkers, Surfactant Protein A2, Aquaporin-5, ELISA, molecular forensics

## Abstract

**Background/Objectives**: Establishing drowning as the cause of death remains one of the most challenging tasks in forensic pathology, particularly when classical autopsy findings are absent, nonspecific, or compromised by decomposition. Molecular biomarkers may provide objective adjunctive evidence in such cases. This study investigated the diagnostic utility of Aquaporin-5 (AQP5) and Surfactant Protein A2 (SP-A2) measured in renal tissue as potential biomarkers of freshwater drowning and evaluated the influence of different freshwater environments on their expression. **Methods**: A controlled experimental study was performed using 29 adult Wistar rats allocated to freshwater drowning groups (Boračko Lake, *n* = 11; Jablaničko Lake, *n* = 11) and non-drowning control groups subjected to mechanical asphyxia followed by one hour of postmortem immersion (*n* = 7). Kidney tissues were collected within a maximum of 5 min after death and analyzed for AQP5 and SP-A2 concentrations using enzyme-linked immunosorbent assay (ELISA). Group differences were evaluated using Kruskal–Wallis and Dunn’s post hoc tests, while effect sizes were estimated using Cliff’s delta. Receiver operating characteristic (ROC) curve analysis was performed to assess the diagnostic performance of each biomarker in distinguishing drowning from non-drowning deaths. **Results**: AQP5 concentrations differed significantly among groups (χ^2^ = 16.05, *p* = 0.00033), with large effect sizes observed in all pairwise comparisons. However, diagnostic performance was poor, as ROC analysis demonstrated no significant discrimination between drowning and non-drowning cases (AUC = 0.534, 95% CI: 0.314–0.754; *p* = 0.762). In contrast, SP-A2 showed significant intergroup differences (χ^2^ = 6.69, *p* = 0.035) and moderate diagnostic accuracy for drowning identification (AUC = 0.752, 95% CI: 0.567–0.937; *p* = 0.008), with a sensitivity of 77.2% and specificity of 81.8% at the optimal threshold. Notably, AQP5 expression appeared suggestive of environmental modulation, whereas SP-A2 demonstrated greater relevance to the drowning process itself. **Conclusions**: Renal SP-A2 showed moderate diagnostic utility as a supportive biomarker for drowning, whereas AQP5 lacked sufficient discriminatory power for cause-of-death determination. The marked variability of AQP5 across freshwater environments suggests that it reflects environmental modulation rather than drowning-specific biological responses. Although neither marker is suitable as a standalone diagnostic tool, renal SP-A2 may represent a useful adjunct in forensic drowning investigations, particularly when pulmonary tissue is unavailable or unsuitable for analysis. These findings should be considered preliminary and applicable only to freshwater drowning conditions, as saltwater drowning was not investigated in the present study. Further validation in human forensic casework and larger experimental cohorts is warranted.

## 1. Introduction

Establishing drowning as the cause of death remains one of the most complex and controversial challenges in forensic pathology. Recent evidence-based reviews have further emphasized that no individual postmortem finding currently possesses sufficient sensitivity or specificity to establish drowning as the sole cause of death, highlighting the need for complementary molecular approaches [1,2]. Despite advances in autopsy practice, no single macroscopic or microscopic finding is considered pathognomonic for drowning, and many classical signs, including pulmonary edema, frothy fluid in the airways, and waterlogged lungs, may also occur in other forms of asphyxia or develop as postmortem artifacts [3,4,5,6,7]. The diagnostic difficulty is further exacerbated in cases involving prolonged immersion, advanced decomposition, putrefaction, or environmental degradation, where traditional morphological indicators may be absent, obscured, or significantly altered [5,6,8]. Consequently, considerable efforts have been devoted to the development of molecular approaches capable of supplementing conventional autopsy findings and improving the diagnostic accuracy of drowning investigations [9].

In recent years, forensic research has increasingly focused on the identification of molecular biomarkers capable of providing objective and reproducible evidence of specific pathological processes occurring before death. Like recent developments in postmortem interval estimation and forensic diagnostics, where immunohistochemical and molecular approaches have significantly improved the interpretation of postmortem biological changes, biomarker-based methodologies offer promising opportunities for improving the diagnosis of drowning [10,11]. The growing integration of molecular pathology into forensic investigations reflects the need for complementary tools capable of overcoming the limitations of conventional autopsy findings [10]. Recent studies have further demonstrated the increasing application of molecular methodologies, including PCR- and qPCR-based approaches, for drowning diagnosis, reflecting a broader shift toward objective biomarker-supported forensic investigations [12].

Among the most promising candidates are biomarkers involved in water transport, hypoxic injury, and pulmonary homeostasis. In particular, aquaporins and surfactant proteins have attracted increasing attention because drowning induces profound disturbances in respiratory function, gas exchange, fluid regulation, and systemic oxygen homeostasis [13,14,15,16,17]. Most available studies, however, have focused predominantly on pulmonary tissues, where the primary pathophysiological consequences of drowning occur. Although such investigations have generated encouraging results, their practical applicability may be limited in medico-legal settings in which lung tissue is severely autolyzed, mechanically damaged, or unavailable for evaluation [8,13,14,15,16,17].

The kidney represents an attractive alternative target for forensic biomarker investigations. Previous forensic studies have demonstrated that aquatic molecular markers can be detected in closed organs, including the kidney, supporting the concept that extrapulmonary tissues may provide valuable diagnostic information in drowning investigations [18]. Compared with pulmonary tissue, renal tissue generally exhibits a greater resistance to autolytic degradation and often remains suitable for molecular analyses even in cases presenting moderate postmortem alteration [19,20]. Furthermore, the kidney plays a central role in maintaining fluid and electrolyte balance and is particularly sensitive to systemic hypoxia, hemodynamic instability, and osmotic disturbances, all of which are hallmark physiological consequences of drowning [14,19]. Consequently, drowning-related biological responses may extend beyond the respiratory system and be detectable within renal tissue [14,19,20].

Aquaporin-5 (AQP5) is a membrane water-channel protein primarily expressed in alveolar epithelial cells and secretory epithelia, where it regulates transcellular water transport and contributes to fluid homeostasis. Experimental and forensic studies have demonstrated that aquaporin expression can be influenced by hypoxia, osmotic stress, inflammation, and environmental factors associated with immersion deaths [13,21]. Previous forensic studies and systematic reviews focusing on aquaporins have suggested that aquaporin expression patterns may vary according to environmental conditions associated with immersion deaths [13,22]. Although AQP5 is predominantly expressed in pulmonary and secretory epithelial tissues, previous forensic investigations have demonstrated that aquaporin-mediated responses may be detectable in extrapulmonary organs and that renal aquaporins may be influenced by drowning-related environmental and osmotic changes. Moreover, studies investigating intrarenal aquaporin expression have suggested that renal water-channel regulation may respond to different drowning conditions, supporting exploration of the kidney as a potential forensic matrix [23]. Nevertheless, the forensic significance of renal AQP5 remains largely unexplored.

Surfactant Protein A2 (SP-A2) is a member of the pulmonary surfactant protein family and plays a critical role in alveolar stability, host defense, and immune regulation. Drowning causes mechanical and biochemical disruption of the alveolar-capillary barrier, leading to surfactant alterations, epithelial injury, and severe hypoxic stress [24]. Numerous forensic studies have demonstrated altered pulmonary surfactant expression in drowning victims, supporting the potential utility of surfactant-related biomarkers in drowning diagnostics [24,25,26,27]. Although SP-A2 is predominantly synthesized within the lungs, evidence suggests that surfactant proteins may enter the systemic circulation following alveolar injury and may subsequently be detected in extrapulmonary tissues under conditions of increased permeability and systemic stress [24,27]. This raises the possibility that pulmonary injury-associated systemic dissemination of surfactant proteins may result in detectable extrapulmonary signals [28]. Consequently, renal SP-A2 concentrations may potentially provide indirect molecular evidence of drowning-related pulmonary damage, although the biological mechanisms determining renal accumulation and persistence of SP-A2 remain incompletely understood.

Despite increasing interest in molecular forensic diagnostics, relatively little attention has been directed toward the evaluation of drowning biomarkers in extrapulmonary tissues. Most available studies have focused on immunohistochemical analyses of lung tissue, while quantitative investigations of renal biomarkers remain scarce [13,25,29,30]. Consequently, the potential role of kidney tissue as an alternative matrix for drowning diagnostics has not yet been adequately characterized. The present study was not designed to replace pulmonary biomarker investigations, which remain the primary focus of forensic drowning research. Rather, it sought to explore whether renal tissue may provide complementary molecular information in situations where pulmonary tissue is unavailable, severely damaged, or unsuitable for analysis.

Therefore, the present study was designed to investigate the diagnostic performance of renal AQP5 and SP-A2 in a controlled experimental drowning model. Specifically, we aimed to: (i) determine whether renal concentrations of AQP5 and SP-A2 differ between drowning and non-drowning deaths; (ii) evaluate whether different freshwater environments influence biomarker expression; and (iii) assess the forensic diagnostic utility of these biomarkers through receiver operating characteristic (ROC) analysis. The study was intentionally conducted in natural freshwater environments rather than standardized laboratory freshwater because one of its objectives was to explore the potential influence of real-world environmental conditions on biomarker expression. This approach was considered particularly relevant from a forensic perspective, where drowning deaths occur in diverse aquatic environments characterized by substantial physicochemical variability. We hypothesized that drowning would induce measurable alterations in renal biomarker concentrations, with SP-A2 demonstrating greater discriminatory capacity than AQP5, while environmental factors might significantly influence AQP5 expression. By exploring these objectives, we sought to clarify the potential role of renal biomarkers as adjunctive tools for forensic drowning diagnostics.

## 2. Materials and Methods

### 2.1. Study Design

This prospective, randomized, controlled pilot study was designed to evaluate the diagnostic performance of renal Aquaporin-5 (AQP5) ELK1472; ELK Biotechnology Co., Ltd., Wuhan, China) and Surfactant Protein A2 (SP-A2) Rat SPA2 ELISA Kit (ELK5499; ELK Biotechnology Co., Ltd., Wuhan, China) in an experimental freshwater drowning model. In addition, the study investigated whether environmental characteristics associated with two freshwater environments influenced biomarker expression patterns.

Animals were housed in standard laboratory cages measuring approximately 45 × 30 × 20 cm under controlled environmental conditions. Following randomization, rats assigned to the drowning groups were individually submerged in either Boračko Lake or Jablaničko Lake until death occurred. Control animals underwent standardized mechanical asphyxia and were subsequently immersed postmortem in one or other of the freshwater environments for one hour.

All experimental procedures were conducted at the Veterinary Faculty of the University of Sarajevo and complied with internationally accepted standards for animal experimentation and forensic biomedical research [31,32].

### 2.2. Ethical Approval

The experimental protocol was reviewed and approved by the Ethics Committee of the Veterinary Faculty, University of Sarajevo, Bosnia and Herzegovina (Approval No. 07-03-256-2/26).

All procedures conformed to the Principles for the Care and Use of Laboratory Animals and were performed in accordance with institutional regulations regarding animal welfare. Efforts were made to minimize animal suffering and reduce the number of animals used while maintaining adequate statistical power.

### 2.3. Animals and Housing Conditions

Twenty-nine adult Wistar rats (Rattus norvegicus), weighing between 250 and 300 g, were included in the study. Animals were housed under standardized environmental conditions consisting of:

- Ambient temperature: 20 ± 2 °C;
- Relative humidity: 60 ± 10%;
- 12 h light/dark cycle;
- Free access to standard laboratory chow and water.

Prior to experimentation, all animals underwent a 7-day acclimatization period.

### 2.4. Randomization and Experimental Groups

Animals were randomly allocated using a computer-generated randomization sequence into four experimental subgroups according to freshwater environment and mode of death. Two groups underwent freshwater drowning in different lake environments, whereas two control subgroups underwent mechanical asphyxia followed by one hour of postmortem immersion in the corresponding lake.

For statistical analyses, the two control subgroups were pooled into a single non-drowning control group (ND-C; *n* = 7), resulting in three analytical groups: Boračko Lake drowning (BL-D), Jablaničko Lake drowning (JL-D), and non-drowning controls (ND-C) (Table 1).

The postmortem immersion control model was selected to simulate forensic scenarios involving postmortem deposition of bodies into water and to distinguish drowning-related biological responses from immersion-associated changes.

### 2.5. Sample Size Calculation

Sample size estimation was performed a priori using G*Power software (Version 3.1). Assuming an effect size of f = 0.40, a significance level of α = 0.05, and a statistical power (1 − β) of 0.80, a minimum group size of approximately eight animals was considered adequate for detecting biologically meaningful differences among experimental groups.

The final study included twenty-nine animals distributed among two drowning groups (*n* = 11 each) and one pooled non-drowning control group (*n* = 7). Although the control group size was slightly below the target value suggested by the power analysis, all available samples meeting the predefined inclusion criteria were retained for statistical evaluation.

### 2.6. Experimental Drowning and Control Procedures

The experimental protocol was designed to reproduce active freshwater drowning under natural environmental conditions. Despite this approach differs from other experimental drowning models previously reported in the literature, we adopt this model because the primary objective of the present study was to evaluate renal biomarker expression and the influence of different freshwater environments, direct submersion in natural lake water was considered the most appropriate approach for exploratory investigation.

For drowning groups, animals were submerged individually in freshwater obtained directly from the respective lake environment until irreversible cessation of spontaneous respiratory activity occurred. Death was confirmed by absence of respiratory movements and cardiac activity.

For control groups, death was induced through standardized mechanical asphyxia without water aspiration. Following confirmation of death, animals were immersed in one or other of the study lakes for one hour. The one-hour postmortem immersion period adopted for control animals was intended to reproduce a common forensic scenario involving postmortem submersion and to allow differentiation between biological responses associated with active drowning and changes related solely to immersion.

Immediately after death or completion of postmortem immersion, animals were removed from the water and transported for tissue sampling.

### 2.7. Environmental Characterization of Freshwater Sites

To characterize the aquatic environments, selected physicochemical parameters were recorded at the time of sample collection.

Measured variables included:

- Water pH;
- Electrical conductivity;
- Turbidity;
- Weather conditions.

Both lakes were sampled under fully overcast conditions with no precipitation and minimal wind activity. Boračko Lake is characterized by neutral to slightly alkaline water, with a pH generally ranging from 7.5 to 8.2, electrical conductivity between 250 and 350 µS/cm, and very low turbidity, typically below 2 NTU, reflecting its oligotrophic nature and high water clarity. In contrast, Jablaničko Lake typically exhibits a pH of 7.3–8.2, electrical conductivity of approximately 300–380 µS/cm, and turbidity ranging from 2 to 5 NTU under normal conditions, although turbidity may increase following heavy rainfall or seasonal fluctuations in water level.

These measurements were obtained to explore potential environmental influences on biomarker expression.

### 2.8. Renal Tissue Collection

Following retrieval from the water, all animals were transported immediately for necropsy according to a standardized protocol, and tissue collection was performed without intentional delay.

Both kidneys were exposed through a midline abdominal incision. Renal cortical tissue was sampled according to a standardized macroscopic protocol by excising tissue from the cortical region while avoiding the visibly distinguishable medulla. Samples were transferred into sterile collection tubes for subsequent analysis. No histological confirmation of cortical tissue purity was performed.

Samples were coded using anonymized identifiers to ensure blinding during laboratory analyses. Tissues were maintained on ice during transport and processed immediately after collection.

### 2.9. Preparation of Kidney Homogenates

Kidney tissues were mechanically homogenized according to the manufacturer’s protocol for enzyme-linked immunosorbent assay (ELISA) analysis. Tissue concentrations of AQP5 were analyzed using the Rat AQP5 (Aquaporin 5) ELISA Kit (ELK Biotechnology, ELK1472), while SPA-A2 concentrations were determined using the Rat SPA-A2 ELISA Kit (ELK Biotechnology, ELK5499), according to the manufacturer’s instructions.

Homogenized samples were centrifuged to obtain clear supernatants, which were subsequently used for biomarker quantification.

All samples were processed under identical laboratory conditions and analyzed within the same experimental period to minimize analytical variability.

### 2.10. Quantification of AQP5 and SP-A2

Renal concentrations of AQP5 and SP-A2 were measured using commercially available sandwich ELISA kits specific for rat proteins. Sandwich enzyme-linked immunosorbent assays (ELISA) were used to quantify biomarker levels, read on an ELISA reader (BioTek Synergy LX Multimode Reader, USA).

The assays were performed according to the manufacturers’ instructions.

Briefly:

1. Standards and sample aliquots were added to antibody-coated microplates.
2. Biotin-conjugated detection antibodies were applied.
3. Horseradish peroxidase (HRP)-linked avidin was added.
4. Following incubation and washing steps, chromogenic substrate solution was introduced.
5. The enzymatic reaction was terminated using the stop solution supplied with the manufacturer’s ELISA kit, according to the manufacturer’s instructions.
6. Optical density was measured at 450 ± 2 nm using a BioTek Synergy LX Multimode Reader (BioTek Instruments, Winooski, VT, USA).

For AQP5:

- Sensitivity: 0.125 ng/mL;
- Detection range: 0.312–20 ng/mL.

For SP-A2:

- Sensitivity: 0.062 ng/mL;
- Detection range: 0.156–10 ng/mL.

Biomarker concentrations were calculated using standard calibration curves generated according to the manufacturers’ protocols. Several samples yielded concentrations below the lower limit of the manufacturer’s calibration range (0.312 ng/mL): Boračko Lake, 3 samples; Jablaničko Lake, 5 samples; control group, 3 samples. These values were estimated by extrapolation of the standard curve and were included in the statistical analyses for exploratory purposes. Results obtained below the validated quantification range were interpreted with caution.

### 2.11. Blinding Procedures

Laboratory personnel responsible for ELISA measurements and statistical analyses were blinded to group allocation.

All specimens were assigned coded identifiers before biomarker analysis. Blinding during tissue collection was not implemented because the personnel performing the necropsies were directly involved in the experimental procedures and therefore necessarily aware of group allocation. However, all specimens were coded immediately after collection, and subsequent laboratory analyses and statistical evaluations were performed under blinded conditions.

### 2.12. Statistical Analysis

Statistical analyses were performed using SPSS version 25.0 (IBM Corp., Armonk, NY, USA).

Data distribution was assessed using the Shapiro–Wilk test. As biomarker concentrations did not satisfy assumptions of normality, nonparametric analyses were applied.

Continuous variables were expressed as:

- Mean ± standard deviation (SD);
- Median and interquartile range (IQR);
- Minimum and maximum values.

Differences among groups were evaluated using the Kruskal–Wallis test. When significant differences were identified, pairwise comparisons were performed using Dunn’s post hoc test with Bonferroni correction.

To quantify the magnitude of observed effects, Cliff’s delta (d) was calculated with corresponding 95% confidence intervals. Effect sizes were interpreted as negligible (<0.147), small (0.147–0.330), medium (0.330–0.474), or large (>0.474).

To assess the ability of AQP5 and SP-A2 to discriminate drowning from non-drowning deaths, receiver operating characteristic (ROC) curve analysis was performed. For ROC analyses, drowning cases from both lakes were combined into a single drowning category and compared with combined control groups. Diagnostic performance was evaluated by calculating:

- Area under the curve (AUC);
- 95% confidence intervals;
- Optimal cut-off values using the Youden index;
- Sensitivity;
- Specificity;
- Positive predictive value (PPV);
- Negative predictive value (NPV).

A two-tailed *p*-value < 0.05 was considered statistically significant.

### 2.13. Study Workflow

A graphical representation of the experimental workflow is provided in Figure 1. Animals were allocated to freshwater drowning groups associated with Boračko Lake (BL-D, *n* = 11) and Jablaničko Lake (JL-D, *n* = 11), or to non-drowning control subgroups subjected to mechanical asphyxia followed by one hour of postmortem immersion. The control subgroups consisted of four animals immersed in Boračko Lake and three animals immersed in Jablaničko Lake and were subsequently pooled into a single non-drowning control group (ND-C, *n* = 7) for statistical analysis.

Following experimental procedures, kidney tissues were collected and analyzed for AQP5 and SP-A2 concentrations using ELISA. The resulting data were subjected to nonparametric statistical analyses and ROC analysis to evaluate the diagnostic performance of both biomarkers.

## 3. Results

### 3.1. Descriptive Statistics

Descriptive statistics for renal AQP5 and SP-A2 concentrations are summarized in Table 2. The control group comprised seven animals and included specimens obtained from both Boračko Lake (*n* = 4) and Jablaničko Lake (*n* = 3) postmortem immersion controls.

The highest AQP5 concentrations were observed in the Boračko Lake drowning group (BL-D), with a mean value of 0.351 ± 0.233 ng/mL. Lower concentrations were found in the Jablaničko Lake drowning group (JL-D) (0.063 ± 0.013 ng/mL) and in the combined control group (0.104 ± 0.034 ng/mL).

For SP-A2, concentrations showed a narrower distribution across groups. The highest mean concentration was observed in the control group (3.773 ± 0.217 ng/mL), followed by the Boračko Lake drowning group (3.635 ± 0.386 ng/mL) and the Jablaničko Lake drowning group (3.420 ± 0.359 ng/mL).

The distribution of biomarker concentrations across the experimental groups is illustrated in Figure 2 and Figure 3.

### 3.2. Magnitude of Intergroup Differences According to Cliff’s Delta Analysis

To evaluate the magnitude of intergroup differences independently of statistical significance, pairwise comparisons were assessed using Cliff’s delta (Table 3).

For AQP5, all group comparisons demonstrated large effect sizes. The strongest effect was observed between BL-D and JL-D animals (d = 0.951; 95% CI: 0.715–0.992), indicating substantial differences between the two freshwater environments. Comparisons between BL-D and controls (d = 0.642) and between JL-D and controls (d = −0.778) were also characterized by large effects.

For SP-A2, a large effect size was observed between JL-D animals and controls (d = −0.704; 95% CI: –0.917 to −0.176). Comparisons between the two drowning groups demonstrated a moderate effect size (d = 0.457), whereas differences between BL-D animals and controls were small (d = −0.259).

### 3.3. Intergroup Comparisons

Overall group differences were assessed using the Kruskal–Wallis test followed by Dunn’s post hoc analysis with Bonferroni correction (Table 4).

A significant difference in renal AQP5 concentrations was observed among the study groups (Kruskal–Wallis χ^2^ = 16.05, *p* = 0.00033).

Post hoc analysis identified a significant difference between BL-D and JL-D animals (*p* = 0.0002). No significant differences were detected between BL-D and controls (*p* = 0.239) or between JL-D and controls (*p* = 0.075).

Renal SP-A2 concentrations also differed significantly among groups (Kruskal–Wallis χ^2^ = 6.69, *p* = 0.035).

Dunn’s post hoc testing revealed a significant difference between JL-D animals and controls (*p* = 0.032). Comparisons between BL-D and controls (*p* = 1.000) and between the two drowning groups (*p* = 0.307) did not reach statistical significance. This pattern indicates that the observed SP-A2 differences were not uniformly distributed across the two drowning environments, with the strongest separation occurring between JL-D animals and controls. Although the overall trend was consistent with altered SP-A2 expression in drowning, the magnitude of this effect appeared to differ between freshwater environments.

### 3.4. Diagnostic Performance of AQP5 and SP-A2

To assess the ability of each biomarker to distinguish drowning from non-drowning deaths, data from the two drowning groups were combined and compared with the pooled control groups using receiver operating characteristic (ROC) analysis (Table 5).

As reported in Figure 4, ROC analysis demonstrated poor diagnostic performance for AQP5 (AUC = 0.534; 95% CI: 0.314–0.754; *p* = 0.762).

The optimal threshold value (0.061 ng/mL) yielded a sensitivity of 27.7% and a specificity of 100%, corresponding to a Youden index of 0.278.

SP-A2 demonstrated greater discriminatory ability than AQP5, as reflected by its higher AUC value. ROC analysis yielded an AUC of 0.752 (95% CI: 0.567–0.937; *p* = 0.008).

The optimal threshold value (3.759 ng/mL) provided a sensitivity of 77.2% and a specificity of 81.8%, with a Youden index of 0.591. Positive and negative predictive values were 89.4% and 64.3%, respectively.

Overall, SP-A2 demonstrated moderate diagnostic accuracy for differentiating drowning from non-drowning deaths, whereas AQP5 exhibited limited discriminatory capability (Figure 5).

## 4. Discussion

The present study evaluated the diagnostic utility of renal AQP5 and SP-A2 in a controlled experimental model of freshwater drowning. In addition to assessing their ability to discriminate against drowning from non-drowning deaths, we explored whether different freshwater environments influenced biomarker expression. The results revealed a marked divergence between the two biomarkers. While AQP5 demonstrated substantial intergroup variability, its diagnostic performance for drowning identification was poor. In contrast, SP-A2 exhibited moderate discriminatory capacity and emerged as the more promising renal biomarker for forensic drowning diagnostics. Recent reviews of postmortem biomarkers in asphyxial deaths have further highlighted the potential of molecular and biochemical methodologies, while also emphasizing the absence of standardized protocols and the need for validation in forensic practice [33].

AQP5 is a transmembrane water-channel protein involved in fluid transport and osmotic regulation. Owing to its role in maintaining water homeostasis, it has been proposed as a potential marker in drowning investigations, particularly because immersion and water aspiration induce profound disturbances of electrolyte balance and tissue hydration [13,22,29,30].

In the present study, AQP5 concentrations differed significantly among experimental groups, and large effect sizes were observed in all pairwise comparisons. However, these differences were primarily driven by the marked separation between the two drowning environments rather than by differences between drowning and non-drowning deaths. Indeed, when drowning cases were pooled and compared with controls, ROC analysis demonstrated essentially random classification performance (AUC = 0.534).

These findings are consistent with previous forensic studies demonstrating substantial variability in aquaporin expression according to environmental conditions. Frisoni et al. reported differential AQP5 expression in freshwater and saltwater drowning victims, suggesting that osmotic conditions may influence aquaporin regulation more strongly than the mechanism of death itself [13]. Similarly, An et al. demonstrated significant changes in renal AQP2 expression associated with different drowning media, further supporting the concept that aquaporin responses are strongly environment-dependent [22]. Systematic reviews have subsequently concluded that, despite their biological plausibility, aquaporins show considerable variability across tissues, causes of death, and environmental settings, limiting their use as standalone forensic biomarkers [29,30].

The pronounced difference observed between Boračko Lake and Jablaničko Lake animals further suggests that local physicochemical factors may influence renal AQP5 expression. Although the present study was not designed to identify specific environmental determinants, and only two freshwater environments were included, the observed differences in AQP5 expression suggest that environmental characteristics may contribute to aquaporin regulation. Future studies involving multiple aquatic environments and larger environmental datasets will be required to formally assess correlations between physicochemical parameters and biomarker expression. Recent systematic evaluations of drowning biomarkers have similarly suggested that AQP5 is more informative for distinguishing freshwater from saltwater exposure than for differentiating drowning from non-drowning deaths per se [1].

Unlike AQP5, SP-A2 demonstrated both statistically significant group differences and meaningful diagnostic performance. ROC analysis yielded an AUC of 0.752, with sensitivity and specificity values of 77.2% and 81.8%, respectively, indicating moderate accuracy in distinguishing drowning from non-drowning deaths.

SP-A2 belongs to the surfactant protein family and is primarily synthesized by type II pneumocytes. It contributes to alveolar stability and pulmonary innate immunity and is known to undergo significant alterations following alveolar injury, aspiration, and severe hypoxic stress [24,26,27]. Numerous forensic studies have associated alterations in pulmonary SP-A expression with drowning-related pathology. Lee et al. observed markedly increased intra-alveolar SP-A deposition in experimentally drowned animals compared with postmortem immersion controls, suggesting that surfactant alterations may reflect active physiological responses occurring during the drowning process [34]. De Dominicis et al. demonstrated increased pulmonary SP-A immunoreactivity in drowning victims compared with other causes of death, supporting its value as a marker of alveolar injury and surfactant disruption [26]. Similarly, Barranco et al. identified significant alterations of SP-A expression in drowning fatalities and emphasized its potential value as an adjunctive diagnostic marker [25].

The novelty of the present study lies in the evaluation of SP-A2 within renal tissue rather than pulmonary tissue. It should be emphasized that the present study was designed primarily as a diagnostic feasibility investigation rather than a mechanistic study. Consequently, the biological pathways underlying renal detection of SP-A2 cannot be definitively established from our data. The extrapulmonary presence of surfactant proteins likely reflects complex interactions among pulmonary injury, systemic dissemination, endothelial permeability, and renal handling mechanisms, all of which warrant dedicated investigation. To our knowledge, quantitative assessment of renal SP-A2 concentrations using ELISA for drowning discrimination has not been previously reported. Interestingly, renal SP-A2 concentrations were lower in drowning animals than in controls.

While the underlying mechanism cannot be fully established from the available data, several explanations may be considered. These interpretations should be regarded as hypothesis-generating because no direct assessment of circulating SP-A2 levels, renal functional changes, histopathological alterations, or tissue localization was performed in the current study.

First, drowning is characterized by disruption of the alveolar-capillary barrier, surfactant dysfunction, and increased pulmonary permeability. Experimental and forensic studies have demonstrated substantial alterations in pulmonary SP-A expression following drowning-related lung injury, suggesting that surfactant proteins may undergo abnormal redistribution after severe alveolar damage [24,25,26,27,34]. Altered pulmonary release and systemic dissemination of surfactant components may therefore contribute to changes in extrapulmonary SP-A2 concentrations.

Second, systemic hypoxia and hemodynamic instability are well-recognized consequences of drowning and may induce endothelial dysfunction, microvascular injury, and alterations in renal filtration physiology [14,17,19]. Such changes could influence the transport, accumulation, or clearance of circulating surfactant-related proteins within renal tissue.

Third, severe physiological stress associated with drowning may modify protein turnover and clearance pathways. Although direct evidence regarding renal SP-A2 metabolism remains limited, systemic inflammatory activation, oxidative stress, and organ dysfunction occurring during drowning have been shown to alter the distribution and degradation of circulating proteins, potentially affecting extrapulmonary SP-A2 availability [27,35].

These hypotheses remain speculative and warrant dedicated mechanistic investigation. Nevertheless, the observed pattern suggests that drowning induces systemic biological effects extending beyond the lungs and being detectable within the kidney.

A key finding of the study is that statistically significant biomarker differences do not necessarily translate into useful diagnostic performance. This distinction is clearly illustrated by AQP5, which showed significant intergroup differences but poor classification accuracy. Conversely, SP-A2 achieved only moderate group separation but yielded substantially better discrimination of drowning cases.

From a forensic perspective, sensitivity and specificity values of approximately 80% should not be interpreted as sufficient for standalone diagnosis. Drowning remains a multifactorial forensic diagnosis requiring integration of scene investigation, autopsy findings, histopathology, toxicology, and ancillary laboratory tests [3,5,8]. This concept is consistent with recent evidence-based forensic reviews concluding that no single pathological marker presently provides definitive diagnostic certainty for drowning, underscoring the potential value of adjunctive biomarker-based approaches [1]. This integrated approach remains essential because even advanced molecular methods and established tests such as diatom analysis have inherent limitations, highlighting the need for complementary biomarkers rather than single definitive indicators [36]. Nevertheless, biomarkers capable of providing objective molecular evidence may be valuable as supportive tools within a multidisciplinary diagnostic framework. This concept is consistent with recent experimental research demonstrating that molecular responses related to oxidative stress and inflammatory activation can effectively distinguish drowning from postmortem immersion, further supporting the development of biomarker-based forensic approaches [35]. This trend is consistent with recent forensic research exploring plankton- and microorganism-based molecular assays, which have demonstrated the potential utility of objective laboratory markers in drowning investigations [12].

Particularly noteworthy is the use of renal tissue as the analytical substrate. The value of extrapulmonary tissues in drowning diagnostics has also been highlighted by molecular studies demonstrating the detection of aquatic microbial markers in closed organs, including the kidney, reinforcing the potential forensic utility of renal-based analyses when pulmonary findings are inconclusive [18]. One of the major challenges in forensic drowning investigations involves cases with advanced decomposition or prolonged immersion, where pulmonary tissue may be severely degraded. The kidney generally undergoes autolytic degradation more slowly than the lungs and often remains suitable for biochemical and molecular analyses during intermediate stages of postmortem change [19,20]. Consequently, the possibility of identifying drowning-related molecular signatures in renal tissue may provide practical advantages in real-world forensic casework.

### 4.1. Strengths and Limitations

This study possesses several strengths. First, the controlled experimental design minimized confounding variables commonly encountered in human forensic investigations. Second, randomization, blind laboratory analyses, and standardized postmortem procedures reduced potential sources of bias. Third, the study explored a novel extrapulmonary tissue source for drowning biomarker analysis. Finally, the inclusion of two independent freshwater environments allowed evaluation of environmental influences on biomarker expression, an aspect rarely investigated in experimental drowning studies.

Several limitations should also be acknowledged. The sample size, although determined through an a priori power calculation, remains relatively small. The experimental model involved only freshwater environments, limiting extrapolation to saltwater drowning scenarios. Furthermore, only renal tissue was analyzed, preventing direct comparison with pulmonary biomarker expression. Although tissue sampling was restricted macroscopically to the renal cortex, microscopic verification of the absence of medullary tissue was not performed. Therefore, minor inclusion of medullary structures cannot be completely excluded. Quantification was performed exclusively through ELISA, without complementary validation by immunohistochemistry, Western blotting, or molecular analyses. Consequently, although ELISA allowed quantitative assessment of biomarker concentrations, the study could not verify the cellular localization, tissue distribution, or expression patterns of AQP5 and SP-A2 within renal tissue. Multi-platform confirmation would strengthen the biological interpretation of the observed findings. Moreover, several samples yielded concentrations below the lower limit of the manufacturer’s calibration range (0.312 ng/mL). These values were estimated by extrapolation of the standard curve and were included in the statistical analyses for exploratory purposes. Although the observed differences in AQP5 may be related to environmental and physiological conditions surrounding death, these findings should be interpreted with caution. The current study does not allow us to establish a direct causal relationship between environmental conditions and AQP5 expression. Further studies with controlled environmental conditions and standardized tissue sampling intervals are needed to clarify the contribution of environmental factors to changes in AQP5. Differences in the interval between death and tissue retrieval may affect biochemical changes after death and, consequently, AQP5 measurements. Furthermore, there is difference in tissue collection timing between drowned and control animals. While samples from drowning groups were collected immediately after death, control animals underwent one hour of postmortem immersion before necropsy. Although this design was selected to simulate forensic scenarios involving postmortem body deposition in water and to distinguish drowning-related biological responses from immersion-related changes, a potential effect of this limited postmortem interval on biomarker concentrations cannot be completely excluded. Additionally, although the rat model reproduces essential drowning-related physiological responses, it cannot fully replicate the complexity of human drowning, including variable aspiration volumes, underlying diseases, intoxication, or environmental exposure conditions. Finally, the effects of prolonged postmortem interval and advanced decomposition were not investigated and should be addressed in future studies.

### 4.2. Future Perspectives

Future studies should focus on validating these findings in human forensic material and on integrating SP-A2 with other promising molecular markers to create multi-biomarker diagnostic panels. Combining renal biomarkers with lung-specific markers, inflammatory mediators, hypoxia-related proteins, or omics-based approaches may further improve diagnostic accuracy. Additional research should also investigate the mechanisms underlying renal SP-A2 alterations and clarify the environmental factors responsible for AQP5 variability. Moreover, studies should focus on validating these findings with immunohistochemistry, Western blotting, and molecular approaches. Such multi-platform validation would allow confirmation of both protein abundance and tissue localization, thereby strengthening the biological interpretation and forensic applicability of renal drowning biomarkers.

Taken together, the present findings suggest that renal SP-A2 may have value as an adjunctive molecular marker in drowning investigations, whereas AQP5 appears to suggest possible environmental influences rather than drowning-specific biological responses. The identification of informative biomarkers in renal tissue opens new avenues for forensic molecular diagnostics, particularly in cases where traditional pulmonary assessment is limited or unavailable. Finally, future investigations should include a larger number of aquatic environments with systematic measurement of physicochemical parameters to determine whether variables such as pH, conductivity, turbidity, osmolarity, or mineral composition are independently associated with renal AQP5 expression.

## 5. Conclusions

This experimental study demonstrates that renal SP-A2 has greater forensic diagnostic value than AQP5 for differentiating drowning from non-drowning deaths. While AQP5 exhibited marked variability across freshwater environments, its poor discriminatory performance suggests that it primarily reflects environmental influences rather than drowning-specific biological responses. In contrast, SP-A2 showed moderate diagnostic accuracy and may represent a useful adjunctive biomarker when conventional pulmonary findings are unavailable or compromised. However, renal SP-A2 cannot independently establish or confirm drowning as the cause of death and should be interpreted only as supportive evidence within a comprehensive forensic evaluation incorporating scene investigation, autopsy findings, histopathology, toxicology, and other ancillary examinations. Although neither marker is suitable as a standalone diagnostic tool, these findings support the potential role of kidney-based molecular analyses as complementary tools in forensic drowning investigations. Further validation in larger cohorts and human forensic cases is warranted to establish their clinical and medico-legal applicability.

## Figures and Tables

**Figure 1:**
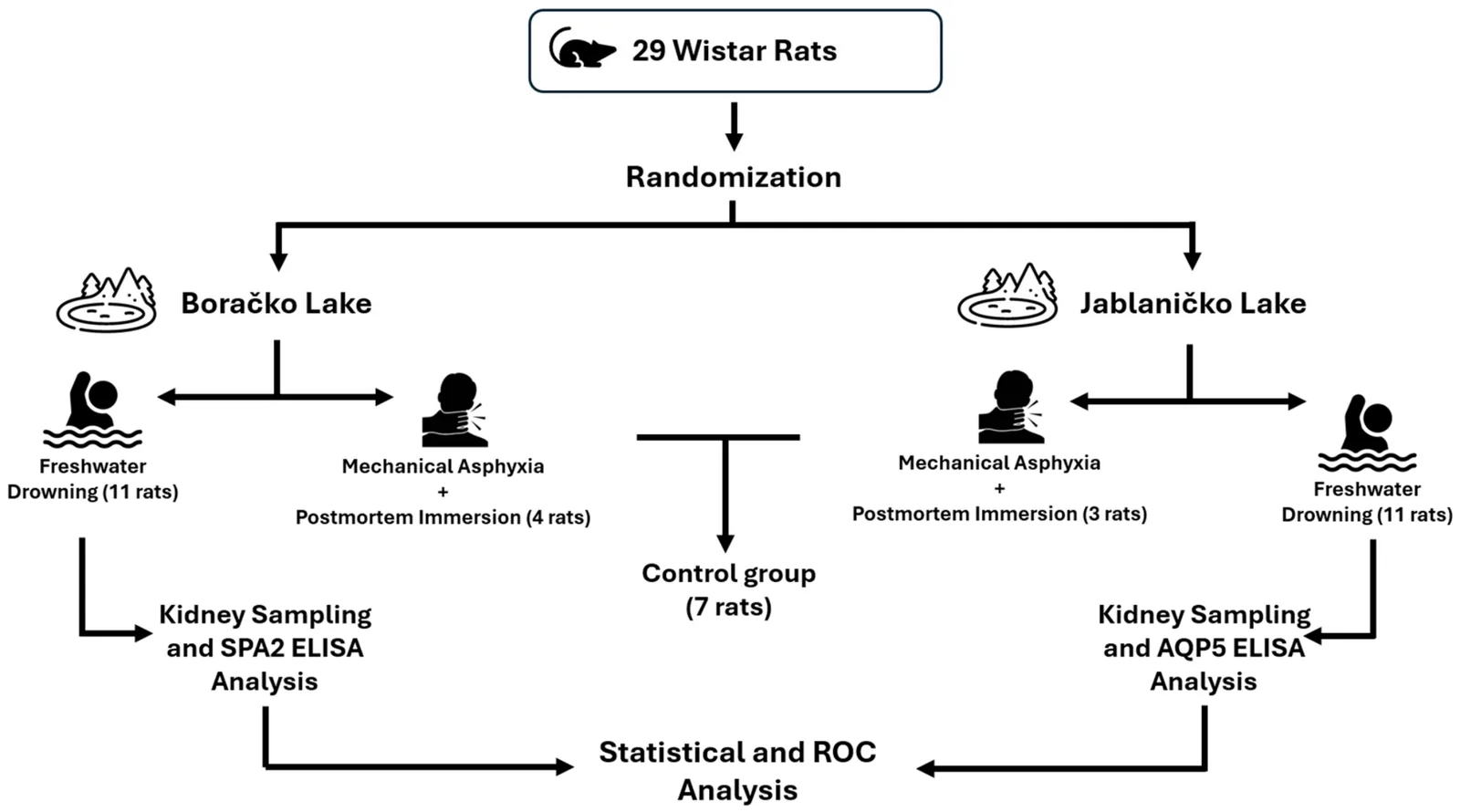
Experimental workflow of the study. Twenty-nine adult Wistar rats were randomly allocated to drowning or non-drowning control groups associated with two freshwater environments (Boračko Lake and Jablaničko Lake). Drowning groups underwent freshwater drowning, whereas control groups were subjected to mechanical asphyxia followed by postmortem immersion. Following death, kidney tissues were collected for biomarker quantification. Renal concentrations of Aquaporin-5 (AQP5) and Surfactant Protein A2 (SP-A2) were measured by ELISA, and the resulting data were analyzed using nonparametric statistical methods and receiver operating characteristic (ROC) analysis to evaluate their diagnostic utility in differentiating drowning from non-drowning deaths.

**Figure 2:**
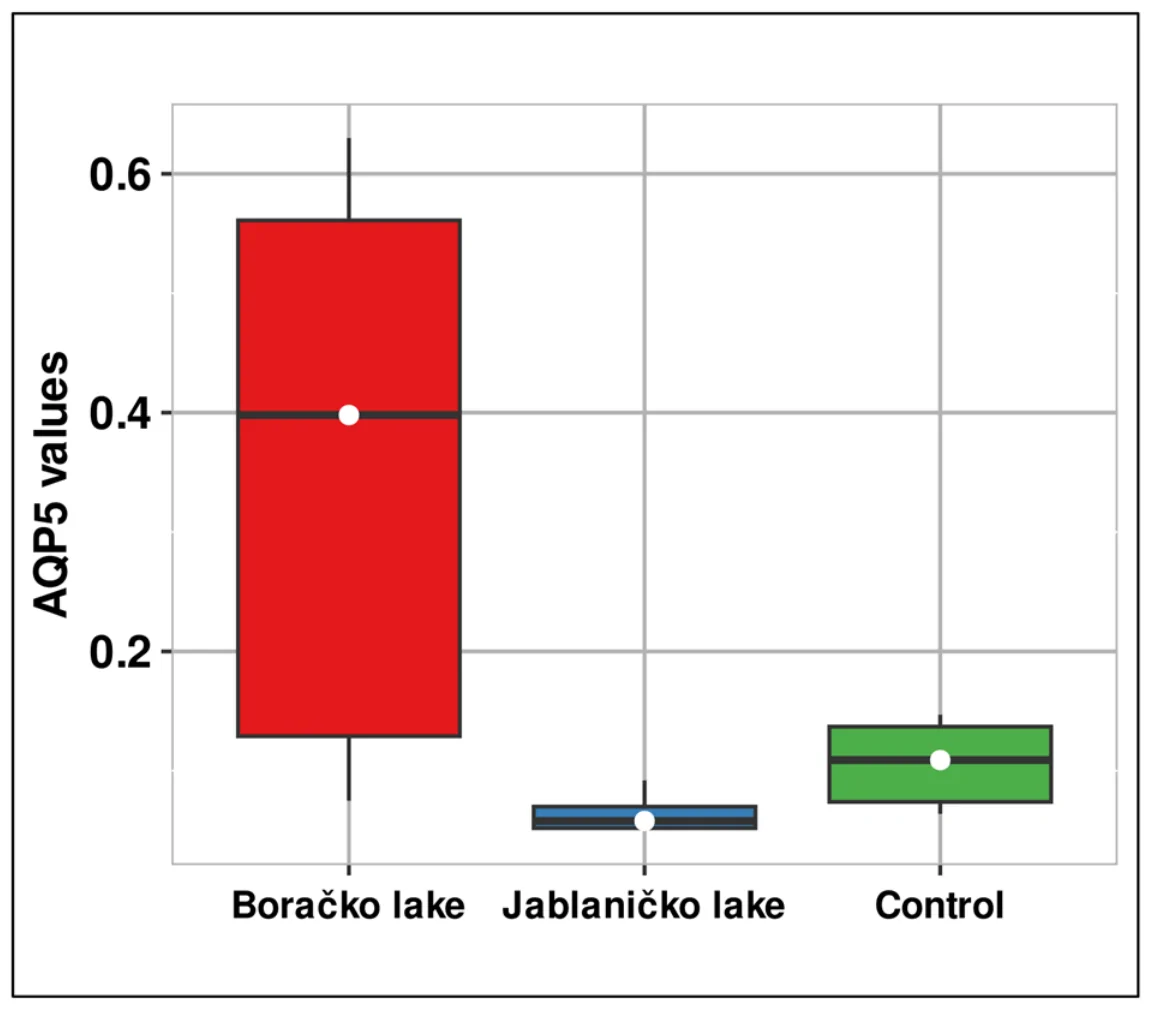
Box-and-whiskers plot for the concentration of AQP5–Boračko Lake–kidney, AQP5–Jablaničko Lake–kidney and AQP5–control group. For the data set, whiskers present the full range of variations (minimum–maximum), boxes present 25th–75th percentiles, horizontal lines in box present median values and white points present mean values (see Table 2).

**Figure 3:**
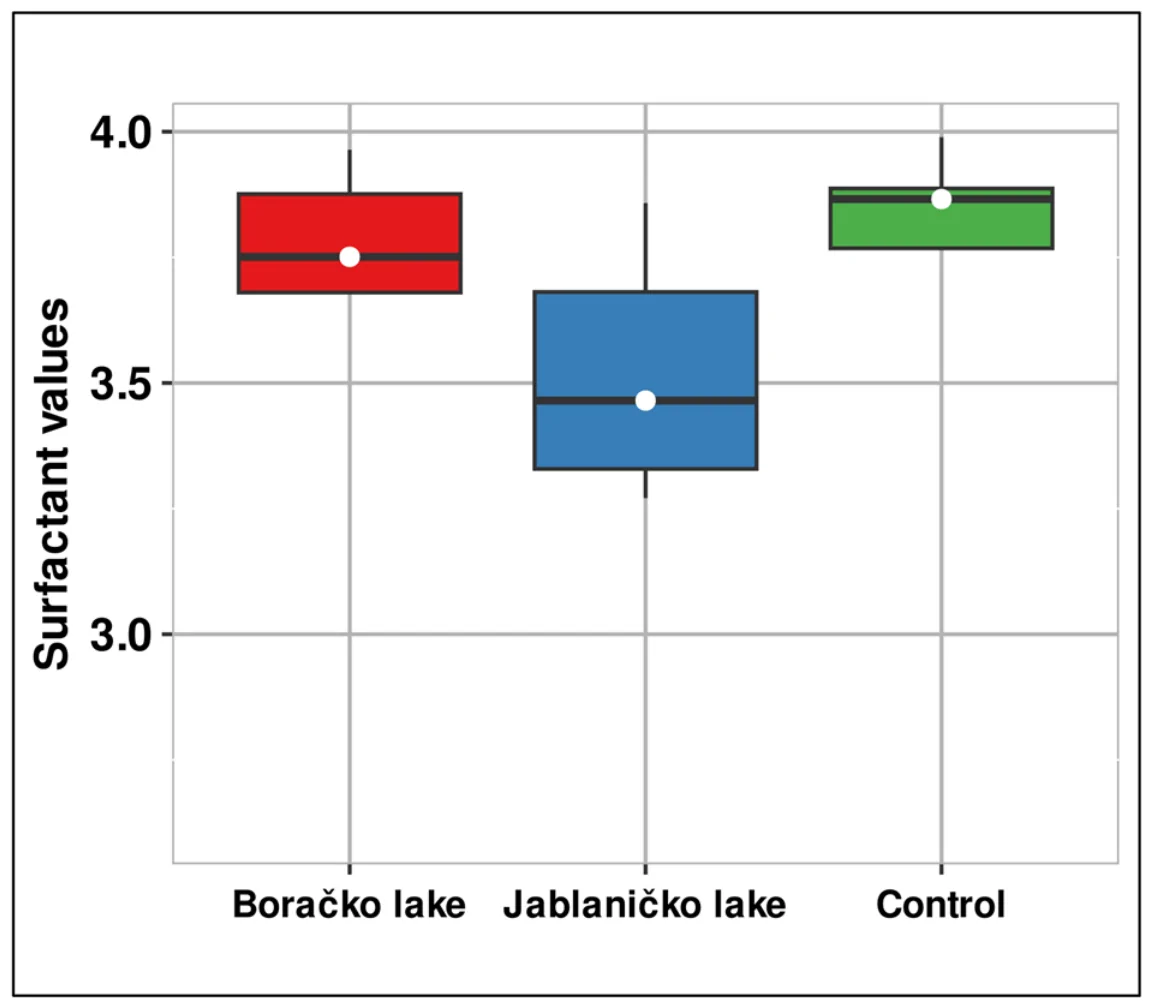
Box-and-whiskers plot for the concentration of surfactant–Boračko Lake–kidney, surfactant–Jablaničko Lake–kidney and surfactant–control group. For the data set, whiskers present the full range of variations (minimum–maximum), boxes present 25th–75th percentiles, horizontal lines in box present median values and white points present mean values (see Table 2).

**Figure 4:**
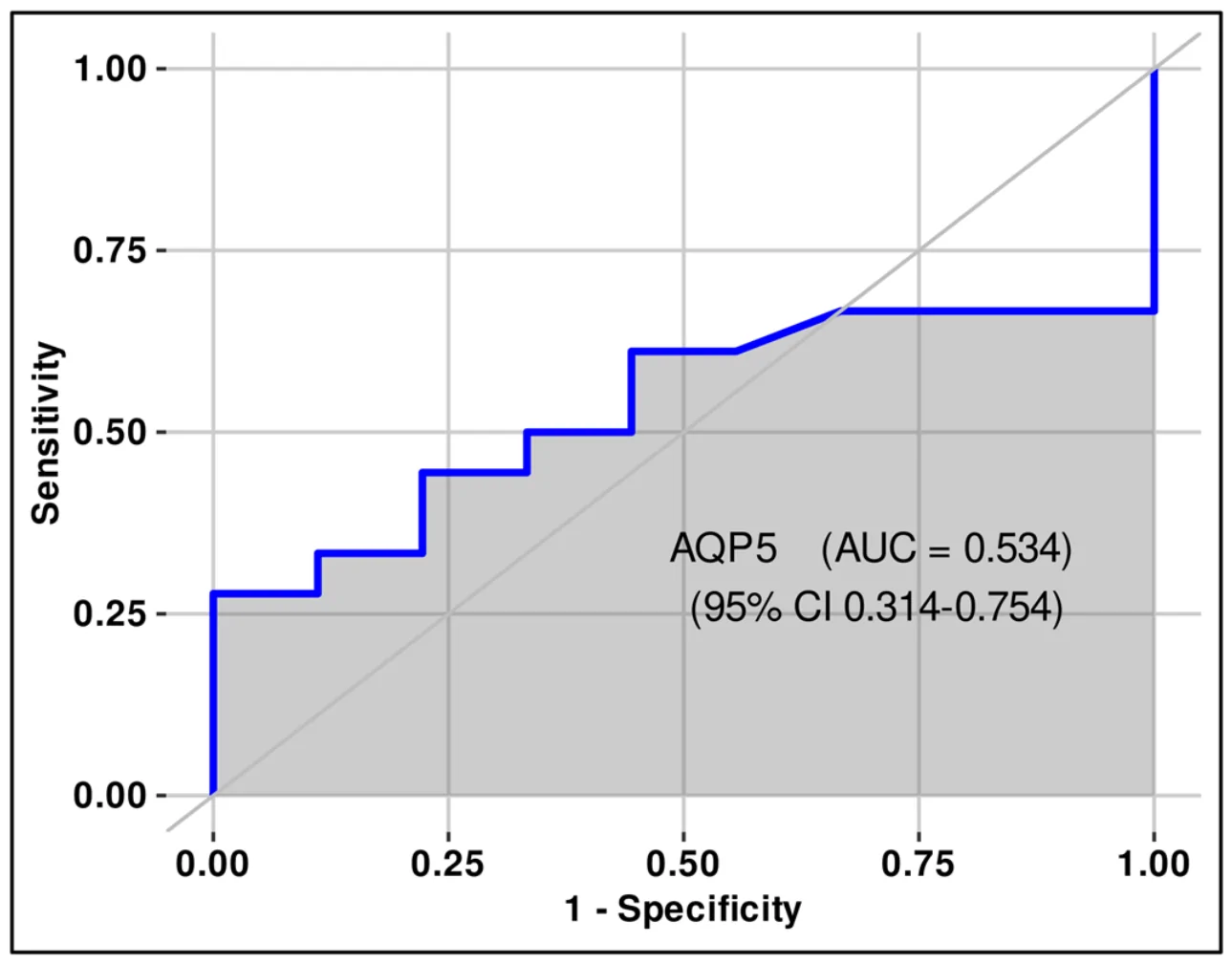
ROC curve for AQP5. The blue line represents the observed receiver operating characteristic (ROC) curve for renal AQP5, while the diagonal reference line represents the line of no discrimination (AUC = 0.5).

**Figure 5:**
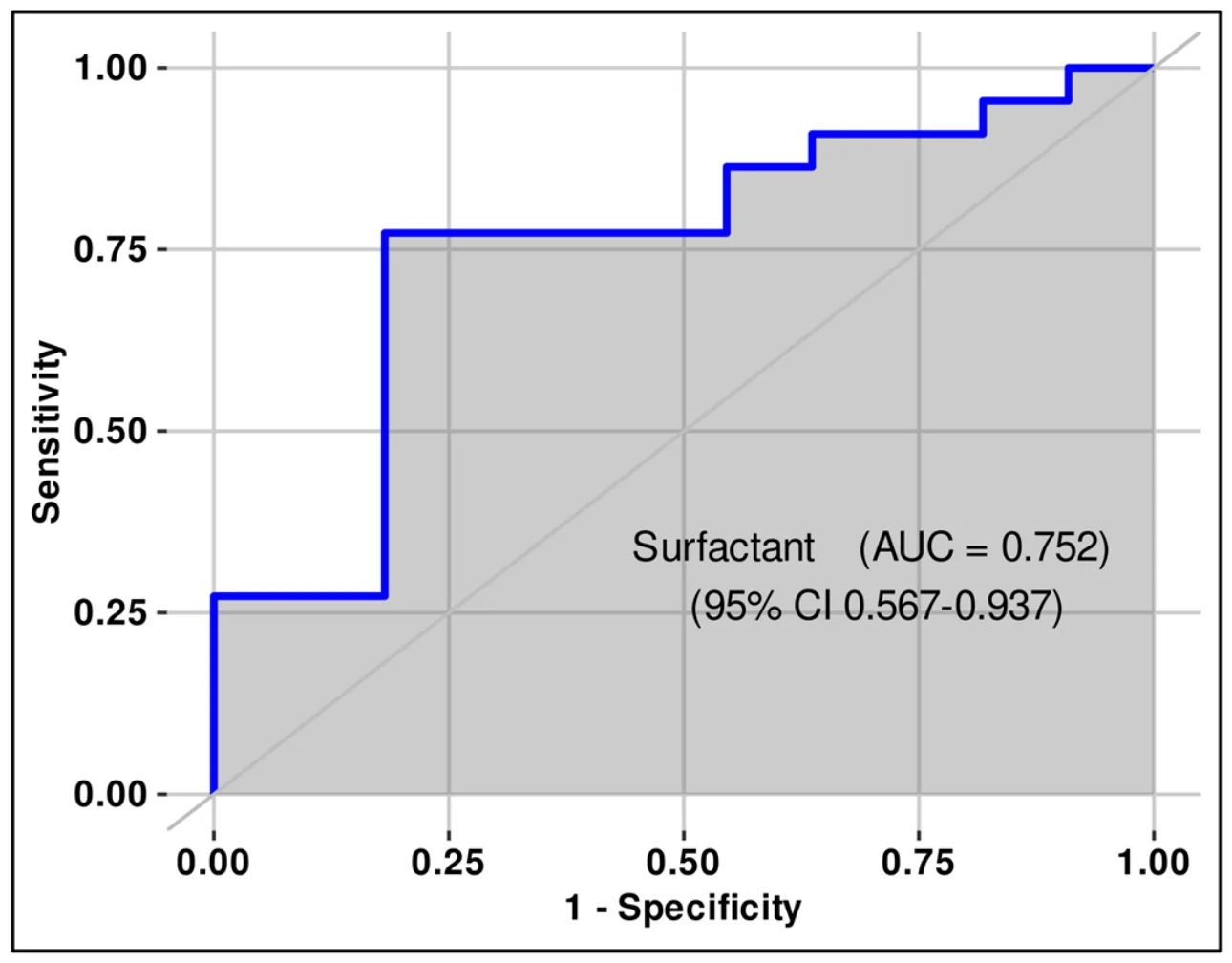
ROC curve for surfactant. The blue line represents the observed receiver operating characteristic (ROC) curve for renal SP-A2, while the diagonal reference line represents the line of no discrimination (AUC = 0.5).

**Table 1:** Experimental allocation of animals and description of study groups. Twenty-nine adult Wistar rats were assigned to drowning or non-drowning control groups according to freshwater environment and mode of death. Drowning groups were subjected to freshwater drowning in Boračko Lake or Jablaničko Lake. The control group consisted of animals subjected to mechanical asphyxia followed by one hour of postmortem immersion (4 animals in Boračko Lake and 3 animals in Jablaničko Lake). The table summarizes group composition, sex distribution, sample size, and experimental procedures.

Group ID	Experimental Group	*n*	Males	Females	Intervention
Group 1:	Boračko Lake Drowning Group (BL-D)	11	6	5	Animals were subjected to freshwater drowning in Boračko Lake.
Group 2	Jablaničko Lake Drowning Group (JL-D)	11	6	5	Animals were subjected to freshwater drowning in Jablaničko Lake.
Group3	Non-drowning Control Group (ND-C)	7	4	3	Animals underwent mechanical asphyxia and were subsequently immersed postmortem in Boračko (4)/Jablaničko (3) Lake for 1 h.

Abbreviations: BL-D, Boračko Lake Drowning Group; JL-D, Jablaničko Lake Drowning Group; ND-C, Non-drowning Control Group.

**Table 2:** Descriptive statistics of renal AQP5 and SP-A2 concentrations across the experimental groups.

Biomarkers–Location–Tissue	Min	Mean ± SD	Median (IQR)	Max
AQP5–Boračko Lake–kidney	0.075	0.351 ± 0.233	0.398 (0.432)	0.630
AQP5–Jablaničko Lake–kidney	0.050	0.063 ± 0.013	0.058 (0.018)	0.092
AQP5–control group–control	0.064	0.104 ± 0.034	0.109 (0.063)	0.147
Surfactant–Boračko Lake–kidney	2.811	3.635 ± 0.386	3.751 (0.196)	3.964
Surfactant–Jablaničko Lake–kidney	2.611	3.420 ± 0.359	3.465 (0.352)	3.858
Surfactant–control group–control	3.388	3.773 ± 0.217	3.866 (0.119)	3.989

**Table 3:** Pairwise effect size estimates (Cliff’s delta) for renal AQP5 and SP-A2 concentrations.

Variable	Cliff’s Delta (d)	Magnitude	95% CI
AQP5–BL–kidney vs. AQP5–JL–kidney	0.951	large	(0.715, 0.992)
AQP5–BL–kidney vs. AQP5–control group–kidney	0.642	large	(0.084, 0.893)
AQP5–JL–kidney vs. AQP5–control group–kidney	−0.778	large	(−0.940, −0.326)
Surfactant–BL–kidney vs. Surfactant–JL–kidney	0.457	medium	(−0.164, 0.818)
Surfactant–BL–kidney vs. Surfactant–control group–kidney	−0.259	small	(−0.690, 0.307)
Surfactant JL–kidney vs. Surfactant–control group–kidney	−0.704	large	(−0.917, −0.176)

JL—Jablaničko Lake; BL—Boračko Lake.

**Table 4:** Kruskal–Wallis and Dunn’s post hoc analyses of renal biomarker concentrations.

Biomarker	χ^2^	*p*-Value	Dunn Post-Hoc Test (Bonferroni)		
				Z	*p*-Value
Surfactant	6.69	0.035	Boračko Lake–Control group	−0.921	1.000
			Boračko Lake–Jablaničko Lake	1.633	0.307
			Control group–Jablaničko Lake	2.554	0.032
AQP5	16.05	0.00033	Boračko Lake–Control	1.753	0.239
			Boračko Lake–Jablaničko Lake	3.996	0.0002
			Control group–Jablaničko Lake	2.243	0.075

**Table 5:** Diagnostic performance of renal AQP5 and SP-A2 for distinguishing drowning from non-drowning deaths.

	AQP5	Surfactant
Area under the ROC curve(AUC)	0.534	0.752
Standard Error	0.112	0.095
95% Confidence interval	0.314–0.754	0.567–0.937
z statistic	0.302	2.665
Significance level *p* (Area = 0.5)	0.762	0.008
Youden index J	0.278	0.591
Associated criterion	0.061	3.759
Sensitivity	0.277	0.772
Specificity	1.000	0.818
Positive predictive value	1.000	0.894
Negative predictive value	0.409	0.643

## Data Availability

Data will be made available on request.
